# Punjab Institute of Neurosciences: Annual Report 2023

**DOI:** 10.12669/pjms.40.12(PINS).11420

**Published:** 2024-12

**Authors:** Asif Bashir, Haseeb Mehmood Qadri, Momin Bashir

**Affiliations:** 1 Asif Bashir, Department of Neurosurgery, Unit-I, Punjab Institute of Neurosciences, Lahore – Pakistan; 2 Haseeb Mehmood Qadri, Department of Neurosurgery, Unit-I, Punjab Institute of Neurosciences, Lahore – Pakistan; 3 Momin Bashir, Department of Neurosurgery, Unit-I, Punjab Institute of Neurosciences, Lahore – Pakistan

## BACKGROUND

The Punjab Institute of Neurosciences (PINS) is one of the largest neurological healthcare facilities in the world, serving a population of over 250 million. Ever since its establishment in 1966 by Professor Bashir Ahmad, one of the pioneers of neurosurgery in Pakistan, it has been providing the best possible neurosurgical care to a vast number of routine and emergency patients suffering from head and spine trauma, brain tumor, and stroke. Originally it operated as the Department of Neurosurgery at King Edward Medical College/Mayo Hospital, Lahore. Due to a shortage of beds at Mayo Hospital, Professor Bashir decided to reestablish the Department of Neurosurgery at Lahore General Hospital (LGH) starting in its old barracks. In a short time, a fully equipped neurological spine center was established that included neurosurgery, neurology, neuroanesthesia, neuroradiology, neuropathology, neuropsychiatry, and neurorehabilitation amongst other fields. Since its inception, the institute has evolved into a regional leader in neurosurgery, now housing 500 beds and pioneering surgical techniques while training generations of neurosurgeons and healthcare professionals. Being the only public sector neurosurgical hospital for almost 60 years, it caters to the patient population from all over Pakistan as well as its neighboring countries.

## STAFF

PINS is currently led by Professor Asif Bashir who is serving as the Executive Director of PINS, Dean of the College of Physicians and Surgeons Pakistan, and member of the Society of Neurological Surgeons, USA. There are three departments of neurosurgery, a dedicated department of neurology, neuropathology, neuroradiology, endovascular neurosurgery, neuroanesthesia, neurorehabilitation and a newly formed department of pediatric neurosurgery. Over 1,000 employees are working at the capacity of PINS that include 102 consultants, 164 postgraduate resident trainees, 393 nursing staff and 360 paramedical and support staff.

## FACILITIES

Despite being a government-funded institution, PINS has acquired state-of-the-art facilities and equipment. As of 2023, these include endoscopes, drills, microscopes, cavitron ultrasonic surgical aspirators (CUSA), neuronavigation systems, stereotactic systems, microelectrodes, three 128-slice CT machines, two 3T MRI machines, and 3D biplane angiography.

## PROCEDURES AND PATIENTS

Between the Departments of Neurosurgery and Neurology, PINS recorded a total outpatient inflow of 266,332 patients in 2023. Close to 10,000 cases are performed per annum with a total of 5,160 procedures performed in the emergency operating theaters and 4,313 procedures performed in the elective operating theaters. In total, 58,613 radiological investigations were performed at PINS (Tables-I, II and III).

**Table-I:** Surgical procedures done in Emergency Operation Theatres at PINS in 2023.

Name of Procedures	Number of Procedures, n	Percentage, % (n/N)
Tracheostomy	1,040	20.15
Extradural hematoma evacuation	642	12.44
External ventricular drain placement	428	8.29
Ventriculoperitoneal shunt placement	403	7.81
Elevation of depressed fracture	451	8.74
Contusion evacuation	409	7.92
Subdural hematoma evacuation	347	6.72
Decompressive craniectomy	226	4.38
Intracerebral hemorrhage evacuation	126	2.44
Brain space occupying lesion excision	105	2.03
Miscellaneous	983	19.05
Total (N)	5,160	100.00

**Table II:** Surgical procedures done in Elective Operation Theatres at PINS in 2023.

Name of Procedures	Number of Procedures, n	Percentage, % (n/N)
Brain space occupying lesion excision	1,452	33.67
Trans-pedicular fixation	486	11.27
Posterior decompression	398	9.22
Discectomy	371	8.60
Aneurysmal clipping	257	5.95
Ventriculoperitoneal shunt placement	245	5.68
Spinal space occupying lesion excision	192	4.45
Anterior cervical discectomy and fusion	92	2.13
Microvascular decompression	89	2.06
CSF rhinorrhoea repair	87	2.01
Transforaminal lumbar interbody fusion	18	0.41
Deep brain stimulation	18	0.41
Miscellaneous	608	14.09
Total (N)	4,313	100.00

**Table-III:** Radiological burden at PINS in 2023.

Neuroimaging	Frequency (n)	Percentage (n/N), %
CT Brain	22,007	37.50
X- rays	11,890	20.30
MRI Spine	9,260	15.80
MRI Brain	7,682	13.10
MRI Brain with contrast	4,715	8.04
CT Spine	1,668	2.85
MRI Spine with contrast	988	1.69
CT Brain with contrast	403	0.69
Total (N)	58,613	100.00

## ACADEMIC ACTIVITIES

The Year 2023 has been a transformative year for PINS academically. The institute established its premier Academic and Research Unit (ARU), Institutional Review Board (IRB) and Audit Review Board (ARB) under Academic and Research Charter (ARC). It initiated a collaboration with the *Pakistan Journal of Medical Sciences* (PJMS), the highest impact factor PubMed-indexed journal in Pakistan.

PINS hosted 24 Grand Rounds featuring national and international educators, neuroscientists, and researchers. It also established an international rotation program for neurosurgery with the University of Missouri, facilitating the bilateral exchange of surgeons and resident trainees. Additionally, PINS presented 6 oral presentations and 12 posters at the 18th World Congress of Neurosurgery, hosted by the World Federation of Neurological Societies (WFNS) in Cape Town, South Africa.

